# Iatrogenic Causes of Cardiac Tamponade Resulting From Surgical Procedures: An Overview

**DOI:** 10.7759/cureus.33773

**Published:** 2023-01-14

**Authors:** Ahmed K Alsenan, Hassan N Al Dhneem, Hussain A Alfandi, Hussain A AlDahan, Hussain M Almadan, Hassan H AlSaif, Ritesh G Menezes

**Affiliations:** 1 Department of Medicine, College of Medicine, Imam Abdulrahman Bin Faisal University, Dammam, SAU; 2 Department of Pathology, College of Medicine, Imam Abdulrahman Bin Faisal University, Dammam, SAU

**Keywords:** malpractice, surgical errors, iatrogenic, medicolegal, cardiac tamponade

## Abstract

Cardiac tamponade is one of the most severely life-threatening emergencies encountered, mainly because of its significant impact on the pumping capacity of the heart by compressing the cardiac chambers due to the rapid accumulation of blood, fluid, pus, or clots in the pericardial sac. These accumulations may be collected following traumas, malignancies, uremia, and many other medical conditions as well as surgical procedures. Numerous errors and medicolegal aspects have been identified in diagnosing and treating cardiac tamponade associated with cardiac-related procedures such as valve replacement surgeries, cardiac pacemaker implantation, pericardiocentesis, and other non-cardiac related procedures such as peri-hiatal surgeries. Patients taking anticoagulants or anticancer medications are especially susceptible to developing cardiac tamponade when undergoing surgical procedures, raising the question of preoperative screening to avoid errors. Misdiagnosis, treatment delay or failure to deliver the utmost quality of treatment, lack of complication screening and follow-ups for those at risk, surgeons rushing to complete cases, burnout, and other human factors are predispositions to the development of cardiac tamponade. Fortunately, most of these errors occurring within healthcare settings are avoidable and must be prevented for eliminating any risks to reduce the incidence and mortality of cardiac tamponade cases resulting from iatrogenic etiology. It is an intricate condition where precision and caution are crucial.

## Introduction and background

Cardiac tamponade is a life-threatening condition due to its significant impact on the pumping capacity of the heart by the pressing force on the cardiac chambers, which is caused by the accumulation of blood, fluid, pus, or clot in the pericardial sac. These accumulations are collected following trauma, effusion, or rupture of the heart [1]. Patients with cardiac tamponade present with tachycardia, pulsus paradoxus, hypotension, muffled heart sounds, and raised jugular venous pressure (JVP). Electrocardiogram (EKG) and echocardiography are needed to diagnose cardiac tamponade. Drainage of the accumulation is the standard of care for the treatment of cardiac tamponade [1]. Cardiac tamponade can be caused by a variety of methods. Studies have shown varying percentages of etiological factors. Strobbe et al. [2] reported the causes of pericardial effusion (88% of the cases were cardiac tamponade) as the following: idiopathic, malignancy, iatrogenic, infections, etc. About 21% of cardiac tamponade causes are iatrogenic, which include surgical and non-surgical causes. A few examples of non-surgical causes, besides malignancies and infections, include treating acute myocardial infarction (MI) with fibrinolytic therapy and other medications such as anticoagulants and anticancer medications. Surgical iatrogenic causes are various and may include cardiac-related procedures such as treating atrial fibrillation with catheter ablation, percutaneous valvuloplasty, pericardiocentesis, pacemaker implantation, and many other interventions [3], as well as non-cardiac procedures following peri-hiatal and diaphragmatic hernia repair surgeries. This manuscript offers an overview of the iatrogenic causes of cardiac tamponade resulting from various surgical procedures and interventions.

## Review

Anatomy of the heart

The heart is an organ located in the chest cavity, specifically the mediastinum. Most of the heart mass is located on the left side in most of the general population, with two axes. The long axis points up to the right shoulder and points down to the apex of the heart, the most inferior and lateral part of the heart [4]. The heart has four chambers, two superior atria, separated from each other by the atrial septum. The two inferior chambers are the ventricles, the bulk of heart muscles, separated from each other by the ventricular septum, and separated from the atria by the atrioventricular septum. The layers of the heart, starting from the inner part, consist of the endothelial layer, myocardial (muscular) layer, and pericardial layer (which also covers part of the major vessels connected to the heart). The pericardial layer is subdivided into two layers, namely, the parietal layer of the pericardium (the outer layer) and the visceral layer of the pericardium (the inner layer), leaving a space between them to form a sandwich cavity, called the pericardial cavity. This cavity normally contains around 50 mL of serous fluid [5]. Pericardial effusion develops when the fluid within the pericardial cavity exceeds 50 mL, which is the normal amount. There are multiple presentations of pericardial effusion, with one being the cardiac tamponade.

Physiology of the heart

The heart is composed of muscles and is considered a muscular pump, pushing blood to all parts of the body. It collects blood from the inferior and superior venae cavae and sends it to the oxygen reservoir, the lung, where gas exchange occurs (takes the inhaled oxygen and leaves carbon dioxide to be exhaled). Subsequently, blood goes back to the heart to distribute to the entire body via arteries originating from the arch of the aorta [6,7].

Pathophysiology of cardiac tamponade

The pathophysiology of cardiac tamponade is understood only after understanding the concept of intrapericardial pressure (IPP). IPP value is below intracardiac pressure and follows all the changes within it. During respiration, IPP changes similar to intracardiac and intrathoracic pressure, so there is no influence on the filling of the heart in normal situations [8]. In case there is effusion in the pericardial cavity, the severity does not solely depend on the amount of accumulated fluid but on the pressure as well, which can be obtained by the pericardial pressure-volume (P-V) relationship. Therefore, in the case of rapid accumulation of 100 mL only, we may have an impact on the IPP leading to hemodynamic instability [9]. Cardiac tamponade occurs in the setting of rapid accumulation in relation to pericardial compliance to raise the IPP above the resting level [10]. This increase in pressure, according to physics, will be transmitted to the intracardiac chambers, mostly on the right atrium, compromising the cardiac fillings (systemic venous-right atrium pressure gradient), developing to a level that the cardiac output cannot maintain systematic and coronary artery perfusion, resulting in cardiovascular collapse.

Procedural errors causing cardiac tamponade

Valve Replacement Surgery

In this procedure, the guidewire uses a pre-valve replacement balloon dilation that may perforate the left ventricle [11], but, primarily, it should not cause perforation in the hands of a cardiovascular surgeon or cardiologist. It might be because of malpractice or unqualified practitioners leading to unintentional patient death.

Percutaneous Aortic Valve Replacement Through Transseptal Mechanism

Cardiac tamponade might be a complication of this procedure. A study published in the Journal of American College of Cardiology found that there is an increase in the mortality associated with this procedure by 7.4% [12], and here is where the role of autonomy and informed consent is highly important in the case of intraoperative complications leading to death, with the family reporting the case to the court to accuse the treating physician, requiring forensics to investigate the case.

Percutaneous Mitral Valvuloplasty

This procedure may cause cardiac tamponade by multiple mechanisms, including perforation to the aortic root and adjacent right atrium due to sliding up of the transseptal set, apical tear by the use of straight-tip balloons accidentally tearing the apex, perforation in the apex by the guiding wires introduced by the catheter to the apex, posterior wall tear of the right ventricle through the dilatation of the track because of septal puncture, and temporary pacing catheter-induced right ventricle perforation [13]. Although many mechanisms lead to cardiac tamponade in this procedure, it does not occur normally. It may be reported to the court of law as malpractice or negligence. In case this procedure leads to death, a postmortem examination may be necessary to investigate the cause of death.

Percutaneous Cardiac Intervention

It can lead to cardiac tamponade, particularly when using guidewire coronary perforation with balloon inflation in the setting of chronic total occlusion [14,15]. One study regarding coronary perforation reported that in the 12 patients who had coronary perforation, three developed cardiac tamponade, necessitating pericardiocentesis. In this case, the cause is clearly iatrogenic, and if it leads to death, forensic confirmation of the cause of death may be required.

Cardiac Ablation Therapy

This procedure requires transseptal puncture along with extensive catheter manipulation and delivering multiple energies to the cardiac chamber wall. It may be complicated by pulmonary vein stenosis, injury to the esophageal wall, thromboembolism, phrenic nerve injury leading to ipsilateral diaphragmatic paralysis, and, more importantly, a significant increase in the incidence of cardiac tamponade [16]. Postmortem examination might reveal the cardiac damage caused by the ablation energy confirming the iatrogenic origin.

Implantation of Cardiac Pacemakers

Pacemaker implantation may lead to cardiac tamponade due to perforation in the cardiac wall. According to a study, it was suspected that 1.7% of 50 patients who underwent pacemaker implantation and presented with chest pain and hypotension within seven days had cardiac tamponade [17]. In another study where all iatrogenic cardiac tamponade patients who required pericardiocentesis or surgical drainage during 1985-2002 collectively, 22 patients were followed up, of whom seven (5.4%) were after pacemaker implantation, three were after temporary pacemaker implantation, and the remaining were after definitive pacemaker implantation [18].

Percutaneous Patent Foramen Ovale Closure

This procedure has major complications such as death, hemorrhage, massive pulmonary embolism, and cardiac tamponade, which occurred in 1.5% of cases in a study that included 1,355 patients [19]. Medicolegal cases might be suspected if the patient died on the table due to negligence of the medical staff in not suspecting cardiac tamponade considering the pathophysiological changes during surgery, requiring a forensic investigation to ascertain the cause of death.

Central Venous Catheter Insertion

Although rare, cardiac tamponade might be due to central venous catheterization causing cardiac perforation, mainly in the right atrium. Cardioverter-defibrillator implantation also increases the incidence of cardiac tamponade due to cardiac perforation. A study from Massachusetts General Hospital included 241 cases of lead implantation and found that in the 130 patients who implanted Riata lead, five (3.8%) developed cardiac perforation compared to none of the remaining 111 patients who implanted sprint fidelis lead. Two of the five patients needed pericardiocentesis for the treatment of cardiac tamponade [20].

Pericardiocentesis

Several case reports have been published delineating cardiac tamponade as a result of pericardiocentesis. Adi et al. reported the case of a 21-year-old young man who presented with shortness of breath and low blood pressure, for which point-of-care ultrasound was done and revealed cardiac tamponade complicating an anterior mediastinal mass. Following saline infusion, the patient continued to deteriorate. Pericardiocentesis was done, and in a couple of hours, the patient succumbed to his illness. The swift removal of pericardial fluid further caused the mediastinal mass to compress the heart chambers, worsening his obstructive shock [21].

Others

Besides cardiac interventions, cardiac tamponade is a complication of many other interventions, such as peri-hiatal surgery, laparoscopic anti-reflux surgery, graft fixation, helical tack, and, most commonly, mechanical hernia repair. These procedures may cause cardiac tamponade through injury to the diaphragmatic dome [22]. Ali et al. reported a distinct case of self-injury that had led to the development of cardiac tamponade. A 33-year-old male was attempting to “pop” a chest cyst with a 14-gauge needle. He became unresponsive and passed away due to the perforation injury causing a build-up of fluid in the pericardium [23].

These etiological factors of cardiac tamponade are important due to the practitioner’s inexperience, malpractice, negligence, or possibly a complication of surgical procedures and can be avoided with more experienced operators. Some surgeons, however, experience cardiac tamponade intraoperatively, which is then managed on-table at the time. Human error, urgency to finish the procedure, and not practically skillful physicians are important factors associated with iatrogenic cardiac tamponade. Therefore, forensic examination may be the main approach to determine if the death of a patient was due to a physician’s mistake where the standards of care were not met, or a complication of surgical procedure where the standards of care were met.

Medicolegal aspects

Medical errors have to be defined appropriately as medical professionals and institutions may fall into the skepticism of whether certain acts are medical errors or not. An acceptable definition of a medical error is the failure to follow the plan of management appropriately or the failure of a surgeon or interventionist, as in our scope, to set up the right procedure of management for a certain aim. Errors can be divided into commission and omission errors. Commission errors are where wrong actions were made, and omission errors are where the appropriate action was not taken [24]. A study by Fernandez et al. reported 143 medicolegal cases of cardiac tamponade, whereby surgical causes were the most common. Having a medicolegal case does not necessarily mean there was a medical error. This might have been an adverse event that the patient was not informed of appropriately. Types of medical errors involved with cardiac tamponade include surgical errors, central line placement, heart valve repair/replacement, and pacemaker/defibrillator were the procedures most commonly associated with medical malpractice [25,26]. Trainees without sufficient education, human factors, rushing to complete cases, and communication gaps between the surgeon and the patient are some of the causes leading to surgical errors [26]. There are risk factors that may result in a surgical error, which should be considered to avoid any surgical error. Distractions, poor staffing, and multiple surgeons performing more than one procedure are examples. Trainee education and their interaction should be addressed sufficiently with appropriate measures in addition to identifying the persons accountable and those responsible for any complications. Adopting a checklist fulfilled by the physicians prior to skin incision may be a preventive measure to ensure patient safety from complications.

Diagnostic errors result in a large number of deaths in healthcare settings, with one in every six patients suffering from a diagnostic error [27]. Common pathways to diagnostic errors are the premature enclosing of the diagnostic process and treatment delay. The most common diagnostic error, however, is seen in primary healthcare settings, wherein there is a failure to order an appropriate investigation, failure to refer, and failure to follow up. An example of a preventive measure for diagnostic errors is when the patient is seen by several healthcare providers of the primary treating team to decrease the probability of a diagnostic error. All surgical errors that may lead to cardiac tamponade mentioned earlier may be subject to a diagnostic error [27].

The main medication errors that are related to cardiac tamponade are anticancer medications and anticoagulants. In fact, it is one of the joint commission patient safety goals to take more time with patients taking these two classes of drugs as they are at a higher risk of developing cardiac tamponade, especially when undergoing surgery [28].

## Conclusions

This review delineates the medicolegal aspects of cardiac tamponade due to iatrogenic surgical causes and further analyzes the situations where surgical errors most commonly lead to the occurrence of cardiac tamponade. Cardiac tamponade may develop as a result of errors from cardiac interventions, such as, but not limited to, valve replacement surgeries, percutaneous valvuloplasty, cardiac pacemaker implantation, and pericardiocentesis, among other procedures such as central venous catheter insertion. Surgeons’ lack of adequate skills, hastiness to finish cases, and communication gaps between surgeons and their patients are factors that may predispose to surgical errors leading to the development of cardiac tamponade. Some patients may have predispositions to developing cardiac tamponade, for example, patients with previous myocardial infarction developing thin myocardium, raising the necessity to screen such patients’ clinical data prior to cardiac interventions. Diagnostic errors are also contributory factors to developing cardiac tamponade such as failure to investigate properly leading to a misdiagnosis, not referring patients to the correct department may hinder the process of management, as well as the lack of follow-ups for patients at risk of developing cardiac tamponade. The avoidance of these errors necessitates establishing a system to rectify healthcare providers’ shortcomings, as well as the process of management so as to not aid treatment delay, surgical errors, or unsuited diagnostic measures. Staff education, especially for the treating physicians and nurses, and the facilitation of tailored communication for medical emergencies is a priority for the primary prevention of cardiac tamponade. Hospitals should thrive to eliminate all iatrogenic factors, including, but not limited to, shortage of staff, poor management tools, and long work hours to prevent surgical errors leading to cardiac tamponade.
